# Optimized Quantification of Fragmented, Free Circulating DNA in Human Blood Plasma Using a Calibrated Duplex Real-Time PCR

**DOI:** 10.1371/journal.pone.0007207

**Published:** 2009-09-28

**Authors:** Martin Horlitz, Annabelle Lucas, Markus Sprenger-Haussels

**Affiliations:** QIAGEN GmbH, R&D Department, Hilden, Germany; Deutsches Krebsforschungszentrum, Germany

## Abstract

**Background:**

Duplex real-time PCR assays have been widely used to determine amounts and concentrations of free circulating DNA in human blood plasma samples. Circulatory plasma DNA is highly fragmented and hence a PCR-based determination of DNA concentration may be affected by the limited availability of full-length targets in the DNA sample. This leads to inaccuracies when counting PCR target copy numbers as whole genome equivalents.

**Methodology/Principal Findings:**

A model system was designed allowing for assessment of bias in a duplex real-time PCR research assay. We collected blood plasma samples from male donors in pools of 6 to 8 individuals. Circulatory plasma DNA was extracted and separated by agarose gel electrophoresis. Separated DNA was recovered from the gel in discrete size fractions and analyzed with different duplex real-time PCR Taqman assays detecting a Y chromosome-specific target and an autosomal target. The real-time PCR research assays used differed significantly in their ability to determine the correct copy number ratio of 0.5 between Y chromosome and autosome targets in DNA of male origin. Longer PCR targets did not amplify quantitatively in circulatory DNA, due to limited presence of full-length target sequence in the sample.

**Conclusions:**

PCR targets of the same small size are preferred over longer targets when comparing fractional circulatory DNA concentrations by real-time PCR. As an example, a DYS14/18S duplex real-time PCR research assay is presented that correctly measures the fractional concentration of male DNA in a male/female mixture of circulatory, fragmented DNA.

## Introduction

Real-time PCR assays have become a widely used method for absolute quantitation of free circulating DNA in plasma or serum samples as the concentration of circulating DNA can be too low for accurate spectrophotometric measurements [1]. Typically, the amplification of one or a few target sequences in a real-time PCR assay is used to quantify the target DNA molecules by comparing the amplification signal of the unknown sample to a standard curve with known DNA concentration [2]. In the case of circulatory DNA, the concentration of the sample is usually determined by comparing the sample to a standard which is made up of high-quality genomic DNA. The concentration of this quantitation standard is, in most cases, determined by measuring UV absorbance. Circulating DNA in plasma, however, is known to be highly fragmented with a majority of the fragments being shorter than 500 bp [1], [3]. This raises the question if circulatory, fragmented DNA can be accurately quantitated by real-time PCR when high-quality genomic DNA is used as a quantitation reference. DNA fragmentation leads to a lower availability of intact target sequences compared to high quality genomic DNA such that, in a circulatory DNA sample, it may be no longer possible to determine the number of diploid or haploid genome equivalents from the detected number of target sequences.

In spite of these technical challenges, the quantitative analysis of circulating DNA is gaining increased importance as a tool of molecular diagnostics addressing various conditions [1]. Circulating DNA derived from tumors can be detected in plasma or serum and the concentration of a tumor-specific target region (e.g., a mutation or a tumor-specific methylation pattern) may reveal details about the malignant condition. Also, measurements of local variations in gene copy numbers can be performend in circulating DNA and the correct measurement of a copy number variation relative to a reference region by real-time PCR hinges on the use of a non-biased quantitative PCR assay. A reliable determination of the quantities of different DNA targets is important as well in the analysis of circulating fetal DNA in the plasma of a pregnant woman. In a situation where a duplex real-time PCR assay or two separate singleplex assays are used to compare abundances of two different target regions in a sample of fragmented DNA, it is crucial that the quantitative detection of the two targets is not influenced by different degrees of degradation of the target DNA sequences—leading to incorrect quantities of the respective target. One example where this may cause biased results is the determination of the proportion of fetal circulating DNA in the total circulating DNA present in maternal plasma where the fetal DNA is detected by a real-time PCR assay specific for a Y chromosome locus (in women carrying a male fetus) and, likewise, total human DNA is measured by targeting an autosomal locus [4]. Correct, non-biased quantification of fragmented DNA is also important in forensic analysis of human DNA. An example is the determination of male DNA content of a case work DNA sample where male and total human DNA are quantitatively detected by a duplex real-time PCR assay in order to determine the percentage of male DNA in the sample [5].

## Materials and Methods

We have tested three different duplex real-time PCR assays for potential quantitation bias in samples of circulatory DNA extracted from plasma of adult, healthy male and female volunteers. Ethics Statement: All blood donation volunteers donated blood samples with informed consent after Review Board approval (Ärztekammer Nordrhein/North Rhine Chamber of Physicians, 40474 Düsseldorf, Germany, case number 2007389). Blood samples were separated by the donor's gender and otherwise processed and stored anonymously.

In a first set of experiments, whole EDTA blood was collected from male donors. Blood samples were obtained by venipuncture and collected into 10 ml EDTA Plus Vacutainer tubes (Becton Dickinson, Heidelberg, Germany). Plasma was prepared within 30 min after blood draw by centrifugation for 10 min at 1800 • g, followed by careful aspiration of the plasma supernatant. Plasma from six to eight individuals was pooled, frozen, and stored below −20°C until further processing. Before DNA extraction, thawed plasma samples were again centrifuged for 15 min at 5500 • g. The plasma supernatant was carefully removed to fresh tubes, kept on ice, and used for DNA extraction within 20 minutes. Circulatory DNA was extracted from 60 mL of plasma using a variation of the QIAamp DNA Blood Midi protocol (QIAGEN GmbH, Hilden, Germany): 10 ml plasma was processed per one QIAamp Mini column using a 20 ml tube extender, 10 ml of buffer AL was used per 10 ml of plasma. After sample lysis, 12 ml of ethanol was added. DNA was eluted in 100 µl buffer EB per column. Eluted DNA from up to six columns was pooled, precipitated with ethanol/sodium acetate, dissolved in 15 µl buffer EB and stored at −20°C until further processing.

DNA concentration of these samples was determined by UV absorbance reading on a NanoDrop spectrophotometer (NanoDrop Technologies, Wilmington, DE, USA).

**Figure 1:**
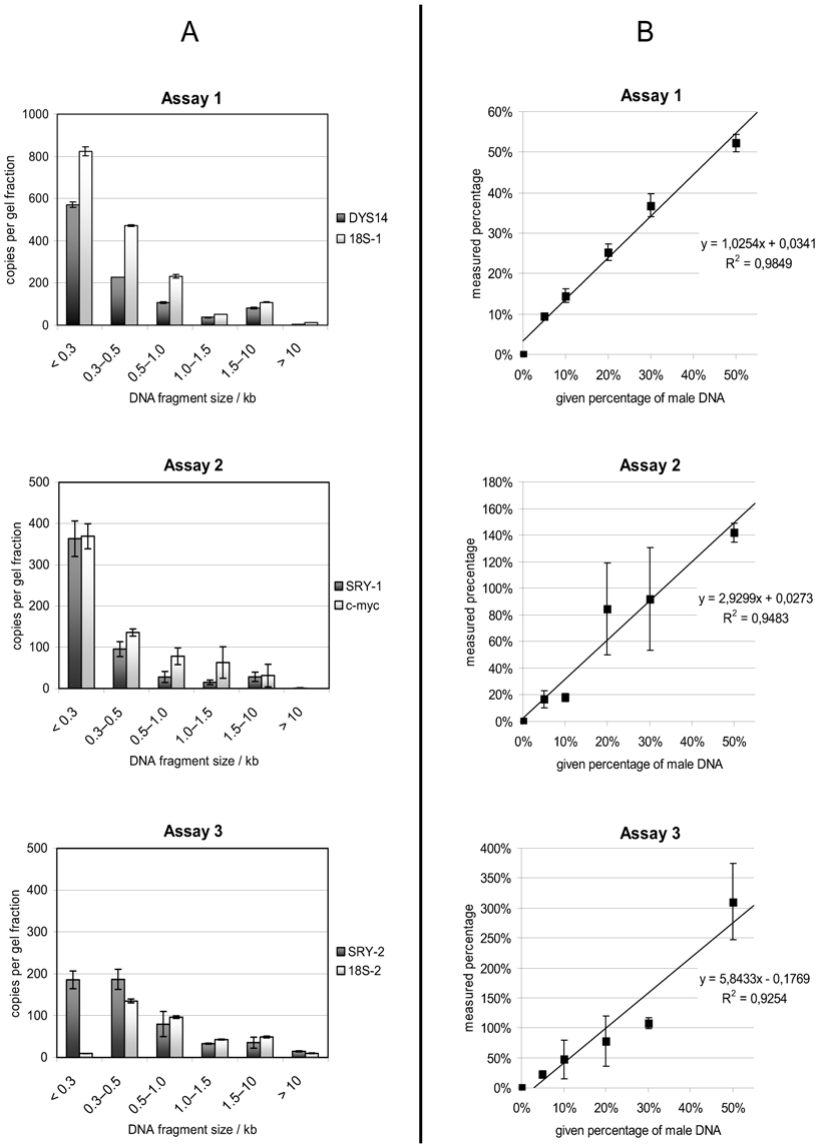
Comparison of three duplex, real-time PCR assays for quantification of circulating plasma DNA. Panel A: Gel-fractionated plasma circulatory DNA from male donors quantified by three different duplex real-time PCR assays. DNA quantities are expressed as haploid genome copies by comparison to a genomic DNA standard curve. Dark columns: Y chromosomal amplicon, light column: autosomal amplicon. The data points represent the mean values +/− one standard deviation calculated from two gel fractionations with four PCR replicates each per DNA fraction (n = 8 data points). Panel B: Mixtures of male and female circulatory DNA. The proportion of male DNA measured by three duplex real-time PCR assays (Y axis) was plotted against the given proportion of male DNA (x axis). Data points represent the mean values of two PCR runs with four replicates per PCR. Error bars correspond to +/− one standard deviation. The curves were fitted using linear regression analysis. The regression curve of an ideal assay, i.e., an assay which measures the male/female DNA ratio correctly, would have the slope b = 1. A significant deviation of the slopes of assay 2 and 3 from the ideal assay was confirmed by statistical analysis (details shown in Supporting Information File 2). The equations and regression coefficients for the curve fits are shown next to the respective plot.

DNA extracted from 10 ml plasma as described above was separated on a 1% agarose gel run at 120–130 mV for 1 h. The whole gel lane from the loading slot to a DNA fragment size of ≈70 bp was cut into slices as described in ref. [2] and as indicated in fig. 1A. The DNA was recovered from the gel slices using the QIAquick gel extraction kit (QIAGEN, Hilden, Germany) according to the manufacturer's handbook and each gel slice was eluted in 100 µl buffer EB (QIAGEN). The DNA in the recovered size fractions was analyzed using different duplex real-time PCR assays in order to check for DNA quantity and the Y-chromosome to autosome ratio.

In a second experiment, circulatory DNA extracted from plasma pools obtained from adult healthy male and female donors, respectively, was mixed to yield female circulatory DNA with a defined proportion of male DNA. The mixtures were subjected to analysis by duplex real-time PCR [6], [7] in order to obtain the measured percentage of male DNA for each given percentage of male DNA. The measured male DNA percentage was plotted against the given percentage and a linear regression analysis was performed with the equation y  =  b • x + a, with y  =  percentage of male DNA (measured), x  =  percentage of male DNA (given), b  =  slope (assay bias), a  =  intercept. The three assays were analyzed for statistically significant differences as shown in Supporting Information File S1.

Real-time PCR using Taqman probes was performed on an ABI7500 instrument (Applied Biosystems, Foster City, CA) targeting the following genes: DYS14 (synonyme for testis-specific protein 1), c-myc (synonyme for v-myc myelocytomatosis viral oncogene homolog), SRY (sex determining region Y), 18S (18S ribosomal RNA cióding sequence). Design of duplex real-time PCR Assays: Assay 1 – DYS14/18S-1 (66 bp/67 bp PCR product size), Assay 2 – SRY-1[8]/c-myc (78 bp/81 bp), Assay 3 – SRY-2/18S-2 (91 bp/187 bp). Further assay and oligonucleotide details are available as Supporting Information File S2.

## Results and Discussion

The size distribution of circulatory plasma DNA separated by agarose gel electrophoresis is shown in fig. 1A: The three duplex assays detected Y-chromosome sequences (SRY, DYS14) and autosomal sequences (c-myc, 18S-1, 18S-2). More than 2/3 of the circulatory DNA present in plasma was of less than 500 bp in size, as detected by SRY and DYS14. Less than 1 % of the fragments were longer than 10 kB. Assay 1 (DYS14/18S-1) showed that the calculated copy numbers for 18S were roughly twice as high as for DYS14 in all fractions. Assay 2 (SRY-1/c-myc) showed a similar pattern, except for the fraction of <300 bp DNA where the copy numbers obtained for both targets were virtually identical. Assay 3 (SRY-2/18S-2) showed a lower copy number for 18S in the fractions <500 bp, so that the ratio of SRY to 18S copies was reversed in those fractions.

The three presented duplex Taqman assays were used to determine absolute quantities of target DNA sequences with interpreting the resulting target copy numbers as representative for a whole genome, i.e. as haploid genome equivalents. The threshold cycle (Ct) values obtained in DNA samples to be measured were converted to copy numbers by comparison to a calibration standard made of male high molecular weight genomic DNA. In cases where the length of the majority of fragments is close to the length of the used real-time PCR target sequences, it follows that a significant part of the target amplicon sequences in the sample is not present as intact, full-length sequence, leading to an under-quantification of the respective target sequence (a model calculation is given in Supporting Information File S3). This can be observed clearly in fig. 1A, Assay 3: In DNA of less than 300 bp, the detected copy numbers for 18S were strongly reduced, leading to a considerable deviation from the correct SRY-to-18S ratio of 1∶2 in fractions of DNA <500 bp. For the SRY/c-myc assay (Assay 2), a similar effect was observed, albeit not as strong. Here, the copy numbers for c-myc appeared to be underestimated in DNA <300 bp when compared to the SRY signal. Assay 1 (DYS14/18S) delivered an unbiased measurement of the DYS14/18S ratio of 1∶2 in all DNA size fractions.

Testing the duplex Taqman assays in the defined male/female mixtures of circulatory DNA (fig. 1B), revealed that Assay 1 estimated the male/female ratio correctly as evidenced by a regression slope b = 1.0. In contrast, the determination of the male/female ratio in both Assay 2 and Assay 3 was significantly different from the expected ratio (as determined by statistical analysis—see Supporting Information File S1) and showed a bias in favor of the Y-chromosome signal. This bias was measured by the slopes of regression curves of Assay 2 (b = 2.9) and Assay 3 (b = 5.8), which indicate a 2.9-fold and 5.8-fold overestimation of the male DNA content in the sample, respectively. It was also apparent that Assay 1 delivers more reproducible estimates of target copy numbers compared to the two other duplex assays as shown by lower standard deviations.

The underestimation of copy numbers in low molecular weight DNA can be explained partly by the different lengths of the target amplicons used. The 18S-1 assay detects a 67 bp target whereas the 18S-2 assay detects a 187 bp target. We expect that in DNA of less than 300 bp, the number of available full-length 187 bp target sequences is reduced to less than 38 % when assuming that the DNA fragments were generated by random cuts (see Supporting Information File S3). This phenomenon has been used previously to determine the lengths of DNA fragments in circulatory DNA by comparing the amplification of targets of different lengths [9], [10]. However, the target sequence length alone does not fully determine the number of available targets in a sample. This suggests the occurrence of non-random DNA cuts in the process of generating the population of circulatory plasma DNA, possibly along nucleosome boundaries. This has been discussed in connection with the proposal that circulatory DNA is generated during apoptosis [11], [12]. A combination of site-specific and random endonucleolytic DNA degradation is likely to generate circulatory plasma DNA. Hence, it is not a given that two different target sequences of the same length produce identical copy numbers in a duplex real-time PCR assay (as evidenced by Assay 2 in our study).

Here we present, with the DYS14/18S-1 assay, a duplex real-time PCR assay using Taqman probes that allows for unbiased quantification of the Y chromosome to autosome ratio in a sample of circulatory DNA prepared from plasma. This was shown in size-fractionated male circulatory DNA and in male/female mixtures of circulatory DNA where the given proportion of male DNA is measured correctly. This assay should also be suitable to quantitate the percentage of male DNA in a forensic sample. Furthermore, this assay could be used to measure the percentage of male fetal DNA in maternal plasma. Using the multicopy DYS14 locus as a real-time PCR target for measuring the amount of fetal DNA in maternal plasma has been reported to be superior to a single-copy locus as SRY in terms of sensitivity and accuracy [13]. Our results confirm these findings. Recently, it has been demonstrated that microfluidics digital PCR provides an advantage over real-time quantitative PCR in terms of accuracy and precision when measuring the fractional concentration of male DNA in male/female DNA mixtures [14]. However, the key experiments of the study used genomic DNA prepared from tissue and blood cells as sample material and as material for quantitation standards and not fragmented circulating DNA prepared from plasma as in the work presented here. It would be of interest to elucidate the performance of microfluidics digital PCR with samples of fragmented circulating plasma DNA in future studies.

Whenever circulating DNA is analyzed, copy numbers derived from a real-time PCR assay with fragmented DNA as a template cannot easily be interpreted as haploid whole genome equivalents because the real-time PCR assay counts only the copies of available full length target. This implies that the detected copy number is, in fact, dependent on the real-time PCR assay performed. Moreover, the target sequences should be as short as possible, as has been shown for the analysis of gene expression levels using partially degraded RNA [15] and CMV viral lead measurements [16]. Therefore, target copy numbers obtained for the same sample using different assays should only be compared after performing appropriate tests that check for quantification bias, e.g. similar to the experiments presented in this paper.

## Supporting Information

Supporting Information File S1 Statistical analysis of results.
(0.03 MB PDF)

Supporting Information File S2 Real-time PCR assay details.
(0.01 MB PDF)

Supporting information File S3 Model calculation of the number of target sequences available for amplification.
(0.01 MB PDF)

## References

[1] Swarup V, Rajeswari MR (2007). Circulating (cell-free) nucleic acids – A promising non-invasive tool for early detection of several human diseases.. FEBS Lett.

[2] Yun JJ, Heisler LE, Hwang, IIL, Wilkins O, Lau SK (2006). Genomic DNA functions as a universal external standard in quantitative real-time PCR.. Nucleic Acids Res.

[3] Li Y, Zimmermann B, Rusterholz C, Kang A, Holzgreve W, Hahn S (2004). Size Separation of Circulatory DNA in Maternal Plasma Permits Ready Detection of Fetal DNA Polymorphisms.. Clin Chem.

[4] Hromandnikova I, Zejskova L, Doucha J, Codl D (2006). Quantification of Fetal and Total Circulatory DNA in Maternal Plasma Samples Before and After Size Fractionation by Agarose Gel Electrophoresis.. DNA Cell Biol.

[5] Nicklas JA, Buel E (2006). Simultaneous Determination of Total Human and Male DNA Using a Duplex Real-Time PCR Assay.. J Forensic Sci.

[6] Hein AE, Bodendorf U (2007). Real-time PCR: Duplexing without optimization.. Anal Biochem.

[7] Ishii T, Sootome H, Shan L, Yamashita K (2007). Validation of universal conditions for duplex quantitative reverse transcription polymerase chain reaction assays.. Anal Biochem.

[8] Zhong XY, Holzgreve W, Hahn S (2001). Risk free simultaneous prenatal identification of fetal Rhesus D status and sex by multiplex real-time PCR using cell free fetal DNA in maternal plasma.. Swiss Med.

[9] Allen Chan KC, Zhang J, Hui ABY, Wong N, Lau TK, Leung TN (2004). Size distributions of Maternal and Fetal DNA in Maternal Plasma.. Clin Chem.

[10] Kiode K, Sekizawa A, Iwasaki M, Matsuoka R, Honma S (2005). Fragmentation of cell-free fetal DNA in plasma and urine of pregnant women.. Prenat Diagn.

[11] Atamaniuk J, Ruzicka K, Stuhlmeier KM, Karimi A, Eigner M, Mueller MM (2006). Cell-Free Plasma DNA: A Marker for Apoptosis during Hemodialysis.. Clin Chem.

[12] van der Vaart M, Pretorius PJ (2007). The Origin of Circulating Free DNA. [Letter].. Clin Chem.

[13] Zimmermann B, El-Sheikhah A, Nicolaides K, Holzgreve W, Hahn S (2005). Optimized Real-Time Quantitative PCR Measurement of Male Fetal DNA in Maternal Plasma.. Clin Chem.

[14] Lun FMF, Chiu, RWK, Chan CA, Leung, TY, Lau, TK, Lo, YMD (2008). Microfluidics Digital PCR Reveals a Higher than Expected Fraction of Fetal DNA in Maternal Plasma.. Clin Chem.

[15] Antonov J, Goldstein DR, Oberli A, Baltzer A, Pirotta M (2005). Reliable gene expression measurements from degraded RNA by quantitative real-time PCR depend on short amplicons and a proper normalization.. Lab Invest.

[16] Boom R, Sol CJA, Schurrman T, van Breda A, Weel JFL (2002). Human Cytomegalovirus DNA in Plasma and Serum Specimens of Renal Transplant Recipients Is Highly Fragmented.. J Clin Microbiol.

