# Supplementary material for: Optimized Quantification of Fragmented, Free Circulating DNA in Human Blood Plasma Using a Calibrated Duplex Real-Time PCR
Source: PLoS One. 2009 Sep 28;4(9):e7207. doi: 10.1371/journal.pone.0007207 (PMC2746311; doi:10.1371/journal.pone.0007207)
Supplement: Supporting Information File S1 — Statistical analysis of results. (0.03 MB PDF) [file pone.0007207.s001.pdf]

Supporting Information File S1

Statistical analysis of results presented in Fig. 1 B:

In order to confirm that Assay 1 estimates the male/female ratio correctly and that Assay 2 and Assay 3 differ significantly from Assay 1, we tested if the regression curve of Assay 1 is coincident (has the same slope and intercept) with the regression curve of Assay 2 or Assay 3. In a first step the variances σ^2 of the three different data sets are tested for equality by computing the residual variances using the following formula:

$$\hat{S}^2 = \frac{1}{n-2} \bullet \sum_{i=1}^{n} \left(y_i - \hat{y}_i \right)^2 \qquad with \quad \hat{y}_i = empirical\ \ regression\ \ line\ \hat{a} + \hat{b} \bullet x_i$$

Table with data sets of the three assays and the calculated residual variances $\hat{S}^2$ (see also Fig. 1 B)

percentage of male DNA	Assay 1 Dys14/18S [%]	Assay 2 SRY/c-myc [%]	Assay 3 SRY/18S [%]
0	0	0	0
5	9.488	16.764	22.517
10	14.523	17.962	47.437
20	25.259	84.667	77.489
30	36.821	92.005	107.761
50	52.284	141.951	310.609
$\hat{S}^2$	24.390	212.520	1654.429

For verifying the null hypothesis that the residual variance of the Assay 1 data set is equal to the residual variance of the Assay 2 or Assay 3 data sets (H_0: $\sigma_1^2 = \sigma_2^2$ or H_0: $\sigma_1^2 = \sigma_3^2$), the two-tailed *F*-Test is used. The test can be carried out by dividing the larger residual variance $\hat{S}_1^2$ by the smaller residual variance $\hat{S}_2^2$:

$$f = \frac{\hat{S}_1^2}{\hat{S}_2^2} \qquad \text{with } (S_1 \geq S_2)$$

Based on the 1-α/2 percentage point in the F-distribution table with $\alpha = 0.05$ (i.e., corresponding to a confidence level of 95%) and $m_1 = n_1 - 2$ and $m_2 = n_2 - 2$ degrees of freedom, the null hypothesis is rejected for $f \geq F_{m_1;m_2;1-\alpha/2}$. In this case: $F_{m_1;m_2;1-\alpha/2}$ = 9.6. The null hypothesis is accepted when $f \leq F_{m_1;m_2;1-\alpha/2}$. The residual variances of the data set from Assay 1 and those from Assay 3 differ significantly because $f \geq F_{m_1;m_2;1-\alpha/2}$ = 67.81 $\geq$ 9.6, whereas the null hypothesis for the comparison of Assay 1 and Assay 2 can be accepted: $f \leq F_{m_1;m_2;1-\alpha/2}$ = 8.71 $\leq$ 9.6.

As Assay 1 and Assay 2 do not differ significantly in terms of their respective residual variances, we tested if the regression coefficients (i.e., slopes) $\hat{b}_1$ and $\hat{b}_2$ of these 2 assays are significantly different. To verify the null hypothesis that $\hat{b}_1$ and $\hat{b}_2$ are equal the following formulas can be used:

$$t_r^{(b)} = \frac{\hat{b}^{(1)} - \hat{b}^{(2)}}{\hat{S}^* \times \sqrt{\frac{1}{(n_1 - 1) \times s_x^2} + \frac{1}{(n_2 - 1) \times s_x^2}}} \qquad \text{with } \hat{S}^* = \sqrt{\frac{(n_1 - 2) \times \hat{S}_1^2 + (n_2 - 2) \times \hat{S}_2^2}{n_1 + n_2 - 4}}$$

Table with calculated values for variances $\hat{S}^2$, $\hat{S}^*$, s_x^2 and slope $\hat{b}$

values	Assay 1	Assay 2
$\hat{S}^2$	24.390	212.520
$\hat{b}$	1.0254	2.9299
$\hat{S}^*$	10.88	
s_x^2	344.167	344.167

Referring to the 1-α/2 percentage point (95% level of confidence), based on $m_1 = n_1 - 2$ and $m_2 = n_2 - 2$ degrees of freedom in the tabulated T distribution, the results (slope) of Assay 2 differ significantly from the results (slope) of Assay 1. The t-value for the comparison of

Assay 1 and Assay 2 is about $t_r^{(b)}$ = 5.13 whereas the $t_{m;1-\alpha/2}$ determined for m = $n_1 + n_2 - 4$ degrees of freedom and a 95% confidence level (1-α/2 percentage point) is about 2.3.

Therefore is $t_r^{(b)} \geq t_{m;1-\alpha/2}$ and the null hypothesis is rejected.

Based on this analysis, it is determined that the results obtained by Assay 2 and Assay 3 differ significantly from the results obtained by Assay 1.
