# Supplementary material for: Optimized Quantification of Fragmented, Free Circulating DNA in Human Blood Plasma Using a Calibrated Duplex Real-Time PCR
Source: PLoS One. 2009 Sep 28;4(9):e7207. doi: 10.1371/journal.pone.0007207 (PMC2746311; doi:10.1371/journal.pone.0007207)
Supplement: Supporting Information File S2 — Real-time PCR assay details. (0.01 MB PDF) [file pone.0007207.s002.pdf]

Supporting Information File S2

Details of real-time PCR reactions:

Real-time PCR reactions were performed using the QuantiTect Multiplex PCR Kit (QIAGEN GmbH, Hilden, Germany) according to the standard recommendations given in the manufacturer's handbook except for the primer and probe concentrations shown below. Independent studies have shown that the used PCR master mix formulation allows for performing quantitative, duplex real-time PCR reactions without optimization to establish equivalence to respective singleplex reactions which are run separately (ref. 6 & 7). The equivalence of real-time PCR results for each duplex assay as compared to the respective singleplex PCR assays was confirmed experimentally (data not shown).

Primers and probes for Assays 1 and 2 were obtained from Operon Biotechnologies, Cologne, Germany

Details of Primers and probes used for real-time PCR:

[In brackets: Concentration used in real-time PCR reactions]

Assay 1, DYS14:

Forward primer: 5'-GAGCAGGCGTGGGTACTATTG-3' [800 nmol/L]

Reverse primer: 5'-GTCTGCTGCTCGGCATCAC-3' [800 nmol/L]

Probe: 5'-CCTGCATGCGGCAGAGAAACCC-3' [200 nmol/L]

Labels: 5' 6-FAM, 3' Black Hole Quencher (BHQ)

Assay 1, 18S-1:

Forward primer: 5’-GCCGCTAGAGGTGAAATTCTTG-3’ [800 nmol/L]

Reverse primer: 5’-CATTCTTGGCAAATGCTTTCG-3’ [800 nmol/L]

Probe: 5’-ACCGGCGCAAGACGGACCAGA-3’ [200 nmol/L]

Labels: 5’ HEX, 3’ BHQ

Assay 2, SRY-1:

Forward primer: 5’-TCCTCAAAAGAAACCGTGCAT-3’ [800 nmol/L]

Reverse primer: 5’-AGATTAATGGTTGCTAAGGACTGGAT-3’ [800 nmol/L]

Probe: 5’-CACCAGCAGTAACTCCCCACAACCTCTTT-3’ [200 nmol/L]

Labeling: 5’ 6-FAM, 3’ BHQ

Assay 2, c-myc:

Forward primer: 5’-CCCTATTCGCTCCGGATCTC-3’ [800 nmol/L]

Reverse primer: 5’-GCCGGCTAGGGTGGAAGA-3’ [800 nmol/L]

Probe: 5’-ACGCCCGCAGCGCAGCTC-3’ [200 nmol/L]

Labeling: 5’ HEX, 3’ BHQ

Sequences taken from ref. (6)

Assay 3:

SRY-2: pre-developed assay from Applied Biosystems, Foster City, CA.

Assay ID: Hs00243216_s1, ref. 4331182

[900 nmol/L primers, 250 nmol/L probe]

18S-2: pre-developed assay from Applied Biosystems, Foster City, CA, ref. 4319413E

[100 nmol/L primers, 250 nmol/L probe]
