# Supplementary material for: Optimized Quantification of Fragmented, Free Circulating DNA in Human Blood Plasma Using a Calibrated Duplex Real-Time PCR
Source: PLoS One. 2009 Sep 28;4(9):e7207. doi: 10.1371/journal.pone.0007207 (PMC2746311; doi:10.1371/journal.pone.0007207)
Supplement: Supporting information File S3 — Model calculation of the number of target sequences available for amplification. (0.01 MB PDF) [file pone.0007207.s003.pdf]

Supporting Information File S3

Model calculation of the number of target sequences available for amplification:

Assumptions for a simplified scenario

(1) DNA fragmentation is assumed to occur as a population of fragments of the same length (i.e., no distribution of fragments of different lengths). This assumption ignores the observed size distribution of DNA fragments in circulatory plasma DNA for the purpose of simplification.

(2) DNA fragments are generated by random endonucleolytic cuts. A possible contribution of site-specific cuts is not considered.

With these assumptions, the proportion p of available (= intact) target sequences in a sample of fragments with length f [bp] and a target with length t [bp] can be calculated as:

$p = 1 - (t/f)$ with $f \geq t$ whereas $f < t$ => $p = 0$

Therefore, the measured number of genome equivalents (GE) can be calculated from the nominal copy number (nominal copy number is defined as the copy number as calculated from the spectrophotometric quantitation of DNA with 3.3 picograms of DNA per haploid genome):

$GE_{measured} = GE_{nominal} * [1 - (t/f)]$

Examples considering the 18S-1 assay (67 bp target) and 18S-2 assay (187 bp target):

f = 10,000 bp	t = 187 bp	=>	p = 98.1 %
f = 1000 bp	t = 187 bp	=>	p = 81.3 %
f = 300 bp	t = 187 bp	=>	p = 37.7 %
f = 200 bp	t = 187 bp	=>	p = 6.5 %
f = 200 bp	t = 67 bp	=>	p = 66.5 %
